# TALEN-mediated knock-in via non-homologous end joining in the crustacean *Daphnia magna*

**DOI:** 10.1038/srep36252

**Published:** 2016-11-07

**Authors:** Takashi Nakanishi, Yasuhiko Kato, Tomoaki Matsuura, Hajime Watanabe

**Affiliations:** 1 Department of Biotechnology, Graduate School of Engineering, Osaka University, 2-1 Yamadaoka, Suita, Osaka, Japan; 2 Frontier Research Base for Global Young Researchers, Graduate School of Engineering, Osaka University, 2-1 Yamadaoka, Suita, Osaka, Japan

## Abstract

Transcription activator-like effector nucleases (TALENs) are versatile tools that enable the insertion of DNA into different organisms. Here, we confirmed TALEN-mediated knock-in via non-homologous end joining in the crustacean *Daphnia magna*, a model organism for ecological and toxicological genomics. We tested two different TALENs, ey1 TALEN and ey2 TALEN, both of which target the eyeless locus. The donor DNA plasmid, harbouring the H2B-GFP reporter gene, was designed to contain both TALEN target sites and was co-injected with each TALEN mRNA into eggs. The ey1 TALEN and ey2 TALEN constructs both resulted in H2B-GFP expression in *Daphnia* with a germline transmission efficiency of 3%. Of the three transgenic animals generated, two had donor DNA at the targeted genomic site, which suggested concurrent cleavage of the injected plasmid DNA and genome DNA. The availability of such tools that are capable of targeted knock-in of foreign genes will be extremely useful for advancing the knowledge of gene function and contribute to an increased understanding of functional genomics in *Daphnia*.

The water flea *Daphnia* is a model organism for ecological and toxicological genomics. This small planktonic crustacean is ubiquitously found in fresh water environments. It has an important position in the aquatic food chain, exhibits a highly plastic phenotype in response to environmental changes, and demonstrates high sensitivity to chemical compounds; such properties have made it a very useful model organism for studies on ecology and toxicology for many decades^1^,^2^. *Daphnia* serves as a useful model organism for understanding reproductive strategies because it normally reproduces asexually but switches between parthenogenesis and sexual reproduction when the environmental quality declines^3^,^4^. In addition, *Daphnia* recently became a model organism for biomedical research at the National Institutes of Health in the United States^5^. Expressed sequence tags (ESTs) and whole genomes have been sequenced for two species (*D. magna*^6^,^7^ and *D. pulex*^8^) and transcriptome analyses have been conducted^9^,^10^. These advancements in *Daphnia* genomics permit the use of this organism as a tool for analysing the genomic response to stressors that could have harmful effects in humans. Because of its multi-faceted role as a model organism in biological research, we chose it for this investigation.

Programmable nucleases are promising tools for targeted gene knock-out and knock-in^11^,^12^. They can be divided into two categories: (i) custom-designed artificial nucleases such as zinc finger nucleases (ZFNs) or transcription activator-like effector nucleases (TALENs), and (ii) RNA-guided nucleases based on clustered regularly interspaced short palindromic repeats (CRISPR)/CRISPR-associated (Cas) system. Both ZFNs and TALENs are fusion proteins, which consist of a programmable DNA binding domain and a *Fok*I nuclease domain. These domains act as a dimer to induce double stranded breaks (DSBs) into the targeted genomic site. The CRISPR/Cas system requires two components, namely programmable single guide RNAs (sgRNAs) and Cas9 nucleases. Single guide RNAs are designed to be complementary to the target genome sequences, whereas Cas9 nucleases induce DSBs at the target site directed by sgRNAs. Repair of the cleaved site, through either the non-homologous end joining (NHEJ) or the homologous recombination (HR) pathways, is a key step for genome editing. In knock-out experiments, NHEJ repair functions through the incorporation of random insertions or deletions (in-dels) at the genomic target site. In knock-in experiments, HR repair has been mainly used for the precise integration of foreign genes via homologous recombination between the genome and donor DNA, which contains the gene(s) of interest. These characteristics have enabled the precise manipulation of the genome in various organisms such as silkworm^13^, cattle^14^, *Brassica oleracea*^15^, *Anopheles gambiae*^16^, *Aedes aegypti*^17^, medaka^18^, liverwort^19^, and wheat^20^,^21^.

In a previous study, we developed a method to inject an exogenous solution into eggs of *Daphnia magna*^22^ and injected TALEN mRNA to assess the corresponding effects^23^. We found that heterodimeric TALENs induced heritable mutations both in the transgene *DsRed2* and in the endogenous eyeless gene, and were associated with decreased lethality compared to homodimeric TALENs. The heritable mutagenesis efficiency of the TALENs was 22% for the eyeless gene, which was more than two-fold higher than that of CRISPR/Cas^23^,^24^. In addition, homologous recombination-mediated knock-in of 67-bp DNA fragments into the eyeless locus was achieved by co-injection of TALEN mRNAs with either single-stranded oligonucleotides (ssODNs) or plasmid DNA harbouring homology arms^25^. However, knock-in of a larger DNA fragment such as a plasmid has not yet been accomplished.

In the current study, using *D. magna*, we tested TALEN-mediated knock-in of plasmid DNA via NHEJ, a method which has been successfully reported in studies using Chinese hamster ovary (CHO) cells^26^, human cell lines^27^, and zebrafish^28^. This knock-in method requires concurrent cleavage of injected plasmid DNA and genome DNA at the targeted site, resulting in the knock-in of exogenous DNA fragments that are much longer than that possible with other approaches, and has the added benefits of simplicity, flexibility, and high efficiency. Our findings show that we successfully integrated a H2B-GFP reporter gene into a targeted genomic locus of *D. magna* demonstrating that TALEN-mediated targeted knock-in via NHEJ can be used in *Daphnia*, and that the availability of this approach will facilitate future efforts towards characterizing and understanding gene function and genomics in this important model organism.

## Results

### Targeted knock-in of *H2B-GFP* into the eyeless locus

**Figure 1:**
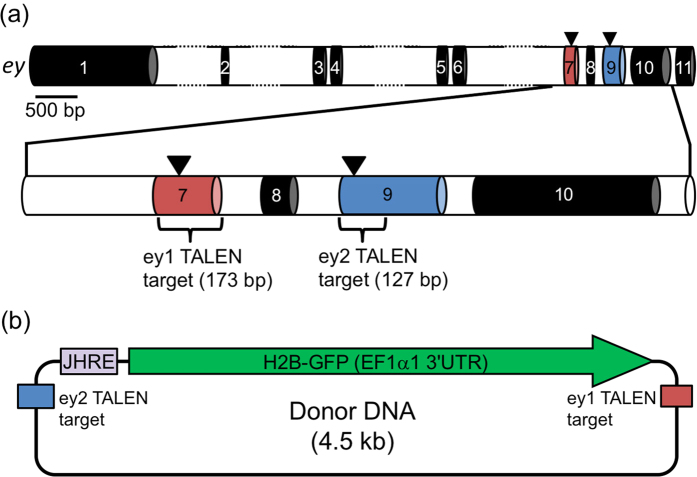
Donor plasmid design for knock-in into the eyeless locus. (**a**) Exon-intron structure of the eyeless (*ey*) gene in *D. magna*. A predicted structure of *ey* is schematically represented based on sequence data obtained from *D. magna* public gene set data and database (http://arthropods.eugenes.org/EvidentialGene/daphnia/daphnia_magna_new/). Exons and introns are shown in black and white respectively, with the exception of ey1 TALEN targeting exon 7 (red) and ey2 TALEN targeting exon 9 (blue). The coding sequence from exon 7 to exon 10 was confirmed experimentally in our previous study^24^. Arrowheads indicate ey1 TALEN and ey2 TALEN cleavage sites. Genomic regions for the insertion of donor DNA by ey1 TALEN target and ey2 TALEN target are shown in brackets. (**b**) Schematic representation of donor DNA. The H2B-GFP ORF attached to *D. magna* EF1a1 3′UTR was used as a reporter. Four tandem repeats of the JHRE element were inserted upstream of the reporter, which was also flanked with the ey1 (red) and ey2 (blue) TALEN target sequences.

To determine whether TALEN achieves targeted knock-in by NHEJ in *Daphnia magna*, we chose the eyeless (*ey*) gene as the target integration site because we previously constructed two different TALEN pairs that target this gene^23^,^25^ (Fig. 1a). Of the two TALEN pairs, ey1 TALEN cleaved exon 7 and introduced biallelic heritable mutations with an efficiency of 42% in addition to insertion of the 67-bp donor insert via homologous recombination using ssODNs and plasmid DNA^25^. Biallelic mutants showed eye deformities because of the gene’s known intrinsic role in eye development. The second TALEN pair, ey2 TALEN, targets exon 9 of *ey* and caused biallelic heritable mutations with an efficiency of 22%^23^. Because of these findings, we chose to use these TALEN pairs in this study.

To confirm genomic integration and germline transmission of plasmid DNA, donor plasmid DNA was constructed to encode a histone H2B-GFP fusion protein with nuclear localization, which facilitates its visualization in individual cells^29^,^30^ (Fig. 1b). We expected juvenile hormone-dependent gene activation during all developmental stages known to occur in arthropod species^31^ and that the resulting knock-in animal could be used for monitoring the activities of juvenile hormone, which is important for *Daphnia* sex determination and phenotypic plasticity in response to environmental stimuli. Therefore, four tandem repeats of the juvenile hormone responsive element (JHRE) from the promoter region of the Krüppel homolog *1* gene from the beetle *Tribolium castaneum*^32^, was inserted upstream of *H2B-GFP.* In addition, the 127 bp and 173 bp regions of the ey2 TALEN and ey1 TALEN target sites were inserted upstream and downstream respectively, in the JHRE and H2B-GFP reporter gene construct.

### Co-injection of TALEN mRNAs with donor plasmid

**Figure 2:**
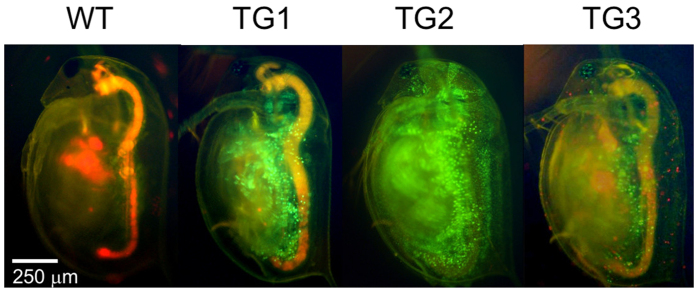
Phenotypes of wild type and transgenic animals. Lateral pictures of wild type (left) and transgenic animals (TG1, TG2, TG3) are shown. In contrast to wild type, broad GFP fluorescence was observed in all transgenic animals.

Firstly, we tested ey1 TALEN-mediated knock-in in *Daphnia*. Donor plasmid DNA was co-injected into 48 eggs with ey1 TALEN mRNAs, which was speculated to lead to concurrent cleavage of the genomic *ey* locus and donor plasmid sequence. Of the 38 surviving animals, one founder produced fluorescent juveniles with normal eyes (Table 1, Fig. 2, TG1). These juveniles were cultured and maintained as a potentially transgenic line (TG1).

Secondly, the activity of ey2 TALEN for knock-in was tested by co-injecting donor plasmid DNA with ey2 TALEN mRNAs into 117 eggs. Of the 60 surviving animals, two founders produced fluorescent juveniles (Table 1, Fig. 2, TG2 and TG3). Similar to TG1, one founder produced juveniles that did not show any eye deformities. The juveniles were cultured and maintained as potentially transgenic line TG2. In contrast, the second founder only produced juveniles with eye deformities. These juveniles had smaller compound eyes that were located at the inner side (Supplementary Data S1), which led to lethality in the offspring before sexual maturation, as has been previously reported^25^. Some offspring from this potential transgenic line (TG3) were collected before death and these underwent genotyping analysis.

### Genotyping of H2B-GFP-expressing *Daphnia*

**Figure 3:**
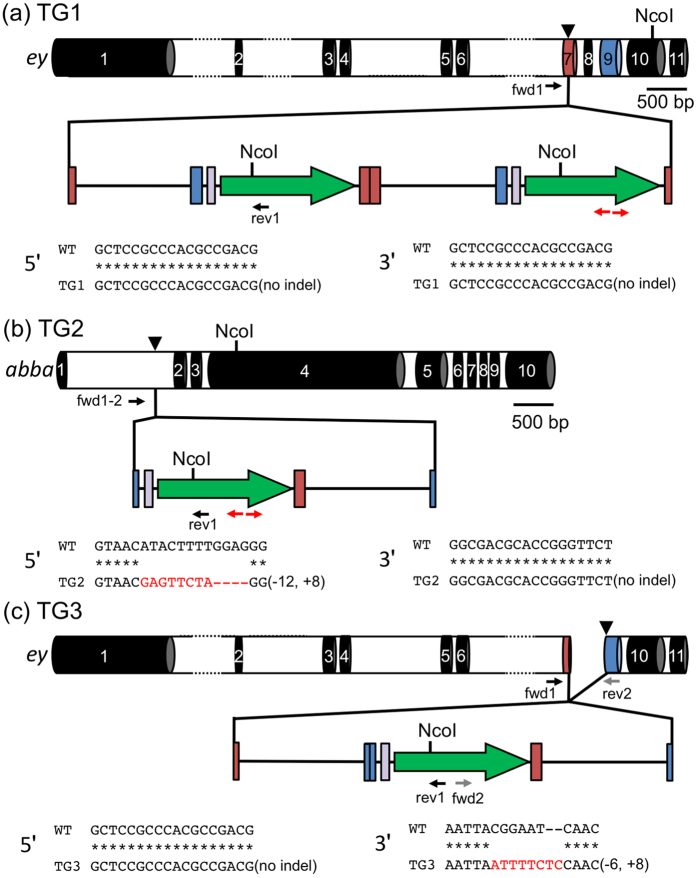
Genotypes of transgenic lines (TG1–TG3). A complete schematic is shown for each of the three transgenic lines produced in this study (TG1, TG2, and TG3). The top image shows the complete structure of the endogenous locus into which donor DNA was integrated. The structure of the integrated plasmid in the genome, and sequences of the junctions between transgene and the surrounding genome are schematically represented at the middle and bottom, respectively. *NcoI* sites used for inverse PCR are shown above each gene. Primer binding sites for inverse PCR are shown with red arrows. Primer positions for genomic PCR to determine 5′ and 3′ junction sequences of the transgene are indicated with black and grey arrows. Red, blue, and grey boxes, and green arrows indicate the ey1 TALEN target, the ey2 TALEN target, the JHRE element, and the H2B-GFP reporter gene, respectively. Arrowheads show the ey1 TALEN and ey2 TALEN cleavage sites.

To identify the integration site of the donor plasmid in potentially transgenic lines TG1 and TG2, we performed inverse PCR followed by genomic PCR and cloned plasmid-to-genomic DNA junction fragments. We found that in TG1 the insertion mapped to the target site of the *ey* locus and that two copies of the H2B-GFP gene were tandemly integrated into the genome. In addition, there were no in-del mutations at the 5′ and 3′ junctions (Fig. 3a). In TG2, we found that the insertion was not mapped to the *ey* locus but to the *abba* (another B-box affiliate) locus and there was a 12-bp deletion and an 8 bp in-del at the 5′ and 3′ junctions, respectively (Fig. 3b).

The lethality of TG3 during juvenile stages prevented us from collecting a sufficient number of animals to obtain sufficient genomic DNA for inverse PCR. Instead, we performed genomic PCR to confirm whether the donor plasmid DNA inserted into the target site of the *ey* locus. The 5′ and 3′ junctions between the transgene and its surrounding genomic region were PCR amplified, cloned, and sequenced. We found tandem integration of two plasmids with a large deletion of the first plasmid at the 5′ junction, whereas the 3′ junction was mapped to the target site of the ey2 TALEN and contained a 6-bp deletion and an 8-bp in-del mutation, suggesting that NHEJ repair occurred at this site (Fig. 3c). Unexpectedly, the 5′ junction was located at the ey1 TALEN-target site in exon 7 and did not have any in-del mutation, although this transgenic animal was created with ey2 TALEN that cleaves exon 9.

## Discussion

The purpose of this study was to test TALEN-mediated knock-in of plasmid DNA in *Daphnia magna* by homology-independent DNA repair. Transgenic animals were established by co-injection of TALEN mRNAs with donor plasmid DNA that contained the TALEN-target sites. This approach enabled the integration of plasmid DNA into the predetermined genomic target site. In *D. magna*, random integration of plasmid DNA by injection occurs with an efficiency of less than 1%^30^. In this study, which used a combination of plasmid DNA and TALEN mRNAs, the efficiency of genomic integration and germline transmission was increased to ~3% (Table 1). In zebrafish, the efficiency of knock-in using NHEJ was reported to be 31 to 50%^28^, which was 6- to 10-fold higher than germline transmission via random integration^33^. The targetable nature and increased efficiency of this approach might significantly advance genome editing of this species.

In this study, the *ey* locus was used as the TALEN-targeted site for knock-in of plasmid DNA, which enabled us to assess the introduction of mutations at this locus by microscopic observation. Of three potential transgenic animals (TG1, TG2, and TG3) that expressed H2B-GFP, TG1 did not show eye deformities. Because genotyping analysis found that one allele of the *ey* locus contained the donor plasmid, the other allele possibly did not have any deleterious mutation. TG2 also produced juveniles with normal compound eyes and the plasmid was found to be integrated into the *abba* locus of these transgenic animals, suggesting that TALEN did not lead to deleterious mutations in both alleles of the *ey* locus. In contrast, TG3 exclusively produced juveniles with deformed eyes and contained donor DNA at one of alleles in the targeted ey locus, suggesting that TG3 harboured deleterious mutations in the other allele. In this line, the transgene was linked at exon 7 without any mutation. Because our donor plasmid contained sequences from exon 7, we hypothesised that the 5′ end of the transgene was ligated via homologous recombination between exon 7 on the plasmid and the endogenous eyeless gene.

Similar to random integration^30^, TALEN-mediated integration of plasmid DNA into the *Daphnia* genome via NHEJ lacked accuracy in terms of the number of DNA copies that were integrated and changes to the sequence of the junction sites between the transgene and the surrounding genomic region. In TG1, the DNA from two plasmids was tandemly integrated into the *ey* locus (Fig. 3a), whereas in TG2 and TG3, in-del mutations were found at the junctions between the transgene and its surrounding genomic region (Fig. 3b,c). It is of note that more precise integration of exogenous DNA might be possible using the recently developed PITCh (precise integration into target chromosome) system, which is based on microhomology-mediated end-joining (MMEJ)^34^,^35^.

An interesting finding from our study was the integration of donor DNA at the untargeted *abba* locus in TG2 (Fig. 3b). There are two possible mechanisms for donor DNA to be integrated into an untargeted locus. One is the off-target integration of the donor DNA. TALEN is known to tolerate a relatively small (<3–4) number of mismatches^36^. However, this untargeted locus contained 5-bp and 11-bp mismatches in the ey2 TALEN-left and ey2 TALEN–right recognition sequences, respectively. In addition, to increase the specificity of TALEN to its recognition site, we used heterodimeric TALENs^23^. Taken together, integration into the *abba* locus did not appear to be a result of TALEN off-target activity. Instead, another mechanism, namely random integration of the donor DNA without TALEN, might have occurred at this locus.

To activate the reporter gene H2B-GFP on the donor plasmid, we inserted four upstream tandem repeats of the JHRE from the promoter region of the Krüppel homolog 1 gene of *Tribolium castaneum*^32^. All transgenic animals showed broad GFP fluorescence, albeit with slight differences in their GFP expression patterns (Fig. 2). Whereas these findings confirm that these JHREs function as activators of gene expression in *D. magna*, it is not known at this time why there are such differences in the expression patterns.

In summary, we demonstrated TALEN-mediated knock-in in *D. magna* through a homology-independent DNA repair method. This approach overcomes limitations of a previous method, which uses only plasmid DNA, including random integration and low efficiency^30^. We believe our genome editing approach might be an important tool that takes advantage of the available EST and genomic sequences to pursue genetic studies in *Daphnia*, and it could lead to a better understanding of the ecology, evolutionary biology, and molecular biology of this important model organism.

## Materials and Methods

### *Daphnia* strain and culture conditions

The wild type *D. magna* strain (NIES clone) was obtained from the National Institute for Environmental Studies (NIES, Tsukuba, Japan) and cultured under laboratory conditions for many generations as described previously^25^.

### Molecular reagents

#### TALEN expression vectors

Two different heterodimeric TALEN pairs, which we previously constructed, were used in this study. One of the TALEN pairs, ey1 TALEN, was pCS-Dmavas-ey-KI-TALEN-left-ELD or pCS-Dmavas-ey-KI-TALEN–right-KKR^25^, whereas the other pair, ey2 TALEN, was pCS-Dmavas-ey-TALEN-left-ELD or pCS-Dmavas-ey-TALEN–right-KKR^23^.

#### Donor plasmid DNA

To generate the donor plasmid, the 4xEcRE region of the 4xEcRE-H2B-GFP plasmid^37^ was replaced with four tandem repeats of the juvenile hormone response element (JHRE) of the Krüppel homolog 1 gene from *Tribolium castaneum*^32^ using the In-fusion HD Cloning Kit (Clontech Laboratories, Inc., CA, USA). The fluorescent reporter gene H2B-GFP encodes a GFP protein fused with *D. magna* histone H2B. The sequence of these four JHRE repeats was 5′-CTCCACGTGATCTCCACGTGTACTCCACGTGCACTCCACGTG-3′. PCR was performed on the genomic regions containing the ey1 TALEN (173 bp) and ey2 TALEN (127 bp) target sites (Fig. 1a). The resulting PCR amplicons of the TALEN target sites were inserted into the donor plasmid (Fig. 1b) using the In-fusion HD Cloning Kit. The primer sequences for amplification of the TALEN targeting sites were as follows: ey1 forward primer (5′-GTGACGATGTGATGTCGGA-3′); ey1 reverse primer (5′-GAGGCTCTCGATCTGTTCG-3′); ey2 forward primer (5′-ACAGGTTTGGTTCAGTAACCG-3′); ey2 reverse primer (5′-CTGCTGCTGGGGATTGAC-3′).

#### *In vitro* RNA synthesis

For TALEN mRNA synthesis, TALEN expression vectors were linearised with *Acc*65I endonuclease, and purified using the QIAquick PCR purification kit (QIAGEN GmbH, Hilden, Germany). Linearised DNA fragments were used for *in vitro* transcription with the mMessage mMachine kit (Life Technologies, CA, USA). Poly(A) tails were attached to TALEN RNAs using a Poly(A) Tailing Kit (Life Technologies), following the manufacturer’s instructions. The synthesised RNAs were column purified using mini Quick Spin RNA columns (Roche diagnostics GmbH, Mannheim, Germany), followed by phenol/chloroform extraction, ethanol precipitation, and resuspension in DNase/RNase-free water (Life Technologies).

### Microinjection

*In vitro* synthesised RNAs and H2B-GFP expression vectors were injected into wild type eggs according to established procedures^30^. Briefly, eggs were collected from wild type daphnids within 2–4 weeks of age just after ovulation and placed in ice-chilled M4 medium containing 80 mM sucrose (M4-sucrose). Synthesised RNA and/or donor DNA were injected using a glass needle and pressurised nitrogen gas. The injection volume was approximately 0.2 nL. Finally, injected eggs were transferred into separate wells of a 96-well plate filled with 100 μL of M4-sucrose. Microinjections were performed within 1 h of ovulation.

### Genomic DNA extraction

A standard phenol/chloroform method for mouse tail DNA extraction was modified and used for this study. Ten adult female daphnids were homogenised using a Micro Smash MS-100 (TOMY, Tokyo, Japan) at 3000 rpm for 1.5 min in 765 μL of lysis buffer (1.18% SDS, 59 mM Tris-HCl, 23.6 mM EDTA, 1118 mM NaCl, pH7.5) followed by the addition of 135 μL of Proteinase K solution (10 mg/mL, Nacalai Tesque, Kyoto, Japan). After an overnight incubation at 50 °C, the homogenate was extracted with phenol and phenol/chloroform/isoamyl alcohol (25:24:1), which was followed by the addition of 2 μL of RNaseA solution (20 mg/mL, Nacalai Tesque). After a 30-min incubation at 37 °C, the DNA solution was further purified with phenol/chloroform/isoamyl alcohol (25:24:1) and chloroform. DNA was precipitated using the addition of an equal volume of isopropanol followed by centrifugation. The pellet was washed with 70% ethanol, briefly dried, and dissolved in TE buffer.

### Inverse PCR

Approximately 2 μg of genomic DNA from transgenic animals was digested with *Nco*I and circularised by ligation for 24 h at 4 °C. Primers used to amplify the plasmid-to-genomic DNA junction were forward (F) 5′-CGGCATGGACGAGCTGTACAAG-3′ and reverse (R) 5′-GGGTGCTCAGGTAGTGGTTGTC-3′. PCR fragments were separated by electrophoresis on an agarose gel, purified, cloned into a pBlunt II-TOPO vector using an In-fusion HD cloning kit (Takara Bio Inc., Shiga, Japan), and sequenced using a Big Dye Terminator v3.1 Cycle Sequencing Kit (Life Technologies) with an ABI 3100 genetic analyser (Life Technologies).

### Genotyping

The targeted genomic regions were amplified by PCR using PrimeSTAR GXL DNA polymerase (Takara Bio, Shiga, Japan). Primers to amplify the plasmid-to-genomic DNA junctions (5′ and 3′) were: 5′ end, fwd1 5′-GTTCCTTTGATCTTTGTTGTCG-3′ and rev1 5′-AACTTCAGGGTCAGCTTGCC-3′; 3′ end, fwd2 5′-CGGCATGGACGAGCTGTACAAG-3′ and rev2 5′-CTGCTGCTGGGGATTGAC-3′, with the exception of forward primer fwd1-2 (5′-ATAGTCCTGGCGGAAATTTC-3′) for amplifying the junction at the 5′ end of TG2. PCR fragments were separated by electrophoresis in an agarose gel, purified, cloned into a pBlunt II-TOPO vector using a Zero Blunt TOPO PCR Cloning Kit (Invitrogen), and sequenced.

## Additional Information

**How to cite this article**: Nakanishi, T. *et al*. TALEN-mediated knock-in via non-homologous end joining in the crustacean *Daphnia magna. Sci. Rep.* **6**, 36252; doi: 10.1038/srep36252 (2016).

**Publisher’s note:** Springer Nature remains neutral with regard to jurisdictional claims in published maps and institutional affiliations.

## Supplementary Material

Supplementary Information

## Figures and Tables

**Table 1:** Summary of knock-in experiments.

Injected mRNAs	Embryos	Juveniles	Adults	Germline transmission
	Injected	Surviving	Surviving	
ey1 TALEN	48	39 (81%)	38 (79%)	1/38 (2.6%)
ey2 TALEN	117	81 (69%)	60 (51%)	2/60 (3.3%)
