# Supplementary material for: TALEN-mediated knock-in via non-homologous end joining in the crustacean Daphnia magna
Source: Sci Rep. 2016 Nov 7;6:36252. doi: 10.1038/srep36252 (PMC5098252; doi:10.1038/srep36252)
Supplement: Supplementary Information [file srep36252-s1.pdf]

Supplementary Information

Title

TALEN-mediated knock-in via non-homologous end joining in the crustacean *Daphnia magna*

Authors

Takashi Nakanishi[1], Yasuhiko Kato[1, 2], Tomoaki Matsuura[1], Hajime Watanabe[1, *]

Affiliations

[1]Department of Biotechnology, Graduate School of Engineering, Osaka University, 2-1 Yamadaoka, Suita, Osaka, Japan

[2]Frontier Research Base for Global Young Researchers, Graduate School of Engineering, Osaka University, 2-1 Yamadaoka, Suita, Osaka, Japan

Corresponding author

Hajime Watanabe[*]

E-mail: watanabe@bio.eng.osaka-u.ac.jp

Tel: +81-6-6879-7427, Fax: +81-6-6879-7428

Contents

Supplementary Data S1

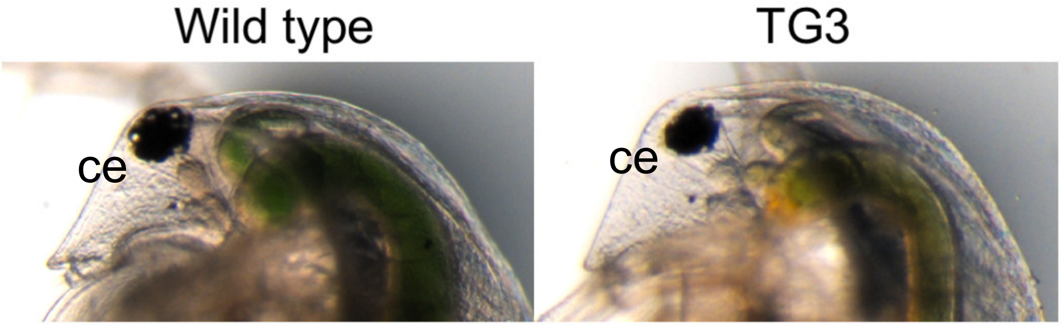

Supplementary Data S1. Phenotype of the transgenic animal TG3

The images to the left and right show the lateral head parts of the wild-type daphniid, knock-in daphniid named TG3 by the TALEN system. Ventral side is left. ce: compound eye.
